# Use of a Novel Chagas Urine Nanoparticle Test (Chunap) for Diagnosis of Congenital Chagas Disease

**DOI:** 10.1371/journal.pntd.0003211

**Published:** 2014-10-02

**Authors:** Yagahira E. Castro-Sesquen, Robert H. Gilman, Gerson Galdos-Cardenas, Lisbeth Ferrufino, Gerardo Sánchez, Edward Valencia Ayala, Lance Liotta, Caryn Bern, Alessandra Luchini, the Working Group on Chagas Disease in Bolivia and Peru

**Affiliations:** 1 Department of International Health, Johns Hopkins University, Bloomberg School of Hygiene and Public Health, Baltimore, Maryland, United States of America; 2 Laboratorio de Investigación en Enfermedades Infecciosas, Universidad Peruana Cayetano Heredia, Lima, Peru; 3 Hospital Universitario Japonés, Santa Cruz, Bolivia; 4 Center for Applied Proteomics and Molecular Medicine, George Mason University, Fairfax, Virginia, United States of America; 5 Global Health Sciences, Department of Epidemiology and Biostatistics School of Medicine, University of California, San Francisco, California, United States of America; Universidade Federal de Minas Gerais, Brazil

## Abstract

**Background:**

Detection of congenital *T. cruzi* transmission is considered one of the pillars of control programs of Chagas disease. Congenital transmission accounts for 25% of new infections with an estimated 15,000 infected infants per year. Current programs to detect congenital Chagas disease in Latin America utilize microscopy early in life and serology after 6 months. These programs suffer from low sensitivity by microscopy and high loss to follow-up later in infancy. We developed a Chagas urine nanoparticle test (Chunap) to concentrate, preserve and detect *T. cruzi* antigens in urine for early, non-invasive diagnosis of congenital Chagas disease.

**Methodology/Principal Findings:**

This is a proof-of-concept study of Chunap for the early diagnosis of congenital Chagas disease. Poly N-isopropylacrylamide nano-particles functionalized with trypan blue were synthesized by precipitation polymerization and characterized with photon correlation spectroscopy. We evaluated the ability of the nanoparticles to capture, concentrate and preserve *T. cruzi* antigens. Urine samples from congenitally infected and uninfected infants were then concentrated using these nanoparticles. The antigens were eluted and detected by Western Blot using a monoclonal antibody against *T. cruzi* lipophosphoglycan. The nanoparticles concentrate *T. cruzi* antigens by 100 fold (western blot detection limit decreased from 50 ng/ml to 0.5 ng/ml). The sensitivity of Chunap in a single specimen at one month of age was 91.3% (21/23, 95% CI: 71.92%–98.68%), comparable to PCR in two specimens at 0 and 1 month (91.3%) and significantly higher than microscopy in two specimens (34.8%, 95% CI: 16.42%–57.26%). Chunap specificity was 96.5% (71/74 endemic, 12/12 non-endemic specimens). Particle-sequestered *T. cruzi* antigens were protected from trypsin digestion.

**Conclusion/Significance:**

Chunap has the potential to be developed into a simple and sensitive test for the early diagnosis of congenital Chagas disease.

## Author Summary

Congenital Chagas disease is one of the main pillars for the control of Chagas disease because 25% of new infections occur by this route. Conventional diagnosis of congenital Chagas disease is based on microscopy at birth and serology at 9 months. However microscopy misses many infections and many at-risk infants fail to complete serology at six to nine months. We have developed a Chagas urine nanoparticle test (Chunap) for concentration and detection of *T. cruzi* antigens. Chunap was evaluated in urine samples of 1-month old children. At this age children have the highest levels of parasitemia and therefore also excrete the highest levels of antigen. Parents prefer a urine test to having their baby's blood drawn. Chunap diagnosed congenital infection in a single urine sample as well as PCR in two blood samples. This study also shows that hydrogel/trypan blue particles used in our test efficiently capture, concentrate and protect urinary *T. cruzi* antigens from enzymatic degradation. Chunap allows for the early diagnosis of congenital Chagas disease, and with appropriate adaptation, may allow early point-of-care intervention.

## Introduction

*Trypanosoma cruzi* is transmitted to humans via vector, organ transplantation, blood transfusion and from mother to fetus [1]. Initiatives to control Chagas disease have achieved remarkable success, as demonstrated by the decrease in estimated prevalence of infected individuals from 20 million in 1990 to 7.8 million in 2005 [2], [3]. Detection of congenital *T. cruzi* transmission is considered one of the pillars of control programs. Congenital transmission accounts for 25% of new infections with an estimate of 15,000 infected infants per year in Latin America [2]–[5]. The earlier in life congenital infection is detected, the higher the efficacy and tolerability of treatment [6]. Current screening programs employ microscopy of fresh blood specimens concentrated by centrifugation in 4 to 6 heparinized capillary tubes (a technique known as the ‘micromethod’) at birth and one month, followed by serology at 6 to 12 months of age [7]–[17]. However, microscopy has low sensitivity (<50% in a single specimen) [7]–[17] and up to 80% of at-risk children fail to complete follow-up in later infancy. Diagnosis is especially problematic in rural areas where tests are not easily accessible or may be poorly performed [8]–[10]. Molecular methods have higher sensitivity than microscopy, but the technical requirements and cost preclude routine use in resource-limited settings. A sensitive, specific and field-friendly screening test is needed to enable effective Chagas disease screening [7]–[9].

Urine antigen detection is an attractive alternative to improve the diagnosis of congenital *T. cruzi* infection. The non-invasive nature promotes high acceptability by parents. Reported sensitivity varies from 32.6% to 100% [18]–[22], depending on the phase of the infection and the methodology used. *T. cruzi* antigens were detected with a sensitivity of 80–90% in urine samples from a small number of congenitally infected infants by a sandwich ELISA test using a panel of monoclonal antibodies [20], [21]; however, this observation was never replicated. In our experience, we achieved a sensitivity of 67.5% in urine ultrafiltrate from acutely infected guinea pigs using a polyclonal antibody to trypomastigote excretory-secretory antigen [19].

Antigens exist in urine in very low concentrations and are susceptible to degradation within minutes after collection. A novel nanotechnology, using capturing nano-porous hydrogel particles produced with poly (N-isopropylacrylamide) (poly(NIPAm)) and N,N′-methylenebisacrylamide (BAAm) and coupled to chemical baits via amidation reaction, has been proposed for concentration and preservation of antigens in urine [23]–[28]. The nano-porous structure performs size sieving, allowing proteins to penetrate inside the particles depending on their molecular weight and shape. The pore size and molecular weight thresholds can be tuned by the percentage of BAAm crosslinker used in the polymerization reaction. The internal chemical baits capture proteins with extremely high affinity (K_D_<10^−12^ M) within minutes [23]–[28]. Chemical baits are covalently bound and distributed throughout the 3-dimensional interior space of the particles yielding a high binding surface and very high binding capacity. Captured antigens can be eluted in a small volume yielding a concentration factor proportional to the volumetric ratio between the initial volume of urine and the final elution volume [23]–[28]. The objective of this study was to evaluate the use of nano-porous particles for sequestration, concentration and preservation of *T. cruzi* antigens in urine, and to apply this technique in urine specimens from congenitally infected and uninfected infants of women with Chagas disease.

## Methods

### Ethics statement

The protocols were approved by the institutional review boards of Hospital Universitario Japones, Asociacion Benefica PRISMA (Lima, Peru), Universidad Peruana Cayetano Heredia (Lima, Peru), Universidad Catolica Boliviana (Santa Cruz, Bolivia) and Johns Hopkins Blooomberg School of Public Health (Baltimore, MD).

### Study design and human samples

The present study is a proof-of-concept study to evaluate the performance of Chunap in the early diagnosis of congenital Chagas disease. Specimens were collected during studies of congenital Chagas disease conducted in Hospital Universitario Japones and Centro de Salud 18 de Marzo in Santa Cruz, Bolivia from 2009 to 2012. The samples included in this analysis represent a select subset (74 specimens from infants without congenital infection and 23 specimens from infants with congenital infection whose urine collection occurred prior to initiation of antitrypanosomal treatment). Urine samples from 12 seronegative infants from Lima, Peru were collected as non-endemic negative controls. Trained study nurses explained the protocol to women presenting for delivery and obtained written informed consent. A specimen was collected to screen for maternal *T. cruzi* infection. The specimen used for screening was a maternal venous blood specimen if labor was not far advanced, or cord blood if the mother was admitted straight to the delivery room in late stages of labor. The screening specimen was tested by 2 rapid diagnostic tests (RDTs): the indirect hemagglutination test (IHA) (PolyChaco, sensitivity and specificity according to manufacturer's instructions: 98% and 99%, respectively) and Trypanosoma Detect, an immunochromatographic strip assay (InBios International) (Sensitivity: 90.7%, Specificity: 100% [29]). A study nurse attended each delivery. For women diagnosed as infected in cord blood, a maternal blood specimen was collected after the mother recovered from the delivery and before discharge from the hospital. Mothers with positive results by one or more rapid tests and their infants were asked to return for follow-up at 1, 6 and 9 months. At the 1-month visit, 5-cc urine samples were collected. Blood samples from infants obtained at 0 and 1 months were evaluated by the micromethod and PCR. The micromethod is the technique used routinely in Bolivian hospitals to screen for congenital Chagas disease. In this technique, cord or neonatal blood is collected in 4–6 heparinized microhematocrit tubes, centrifuged and the buffy coat layer examined microscopically for parasites [30]. Maternal sera and the 6- and 9-month infant sera were tested by at least two of the following IgG serology assays (sensitivity and specificity according to manufacturer's instructions): Chagatest Recombinante ELISA (Wiener Laboratories, Argentina. Sensitivity: 99.3%, and Specificity: 100%), Chagatest ELISA with *T. cruzi* cytoplasmic and membrane antigens (Wiener Laboratories, Argentina. Sensitivity: 100%, and Specificity: 99.6%), and the IHA (PolyChaco). Infants were considered to have congenital infection if they had positive results by microscopy or PCR in 0 or 1 month specimens, or positive results by two or more serologic tests at 6 or 9 months. The nurses were blinded to the Chagas status of each infant. Infants with confirmed infection were referred for treatment by the physicians designated by the Bolivian National Chagas Disease Control Program [31].

### Nano-porous particles synthesis

Hydrogel nano-porous particles were synthesized as previously described [23]–[28]. Briefly, N-Isopropylacrylamide (NIPAm, 0.084 mmol), N,N′-methylene bisacrylamide (BAAm, 0.005 mmol), and acrylic acid (AAc, 0.015 mmol) (Sigma-Aldrich, MO-USA) were dissolved in 1000 mL of MilliQ water and filtered. The solution was purged with nitrogen at room temperature and medium stirring rate for 1 h, and then heated to 70°C. Potassium persulfate (Sigma-Aldrich, MO-USA, 0.002 mmol/ml) was added to initiate the polymerization. The reaction was maintained at 70°C under nitrogen for 6 h. Particles were washed with water five times by centrifugation (19,000 rpm, 50 min). Dye molecules containing an amine group (acid blue 22, acid black 48, bismarck brown Y, pararosaniline base, trypan blue and remazol brilliant blue R) were coupled by condensation to the carboxylic group of acrylic acid present in the poly(NIPAm-co-AAc) particles. Activation of the carboxylic group present in the particles was performed as follows. A 50 mL aliquot of the poly(NIPAm-co-AAc) particle suspension was centrifuged, the supernatant was discarded and the particle pellet was re-suspended in 50 ml of 0.2 M NaH2PO4 pH 5. Five mL of 1% SDS (w/v), 4120 mg of N-(3 Dimethylaminopropyl) N′ ethyl carbodiimide hydrochloride (Fluka Analitical) and 3060 mg of solid N-Hydroxy succinimide (Sigma-Aldrich) were added. The reaction was held at room temperature and medium stirring rate for 15 min. Then, the suspension was centrifuged, and the particle pellet was resuspended in 0.2 M Na_2_HPO_4_ pH>8. The dyes (molar ratio of dye/acrylic acid 10∶1) dissolved in 0.2 M Na_2_HPO_4_ buffer pH>8 were incubated with the activated particles at room temperature and medium stirring rate overnight. After washing steps particles were resuspended in MilliQ water. Once made these particles are stable at room temperature for up to 12 months.

### Nano-porous particle characterization

Dependence of particle diameters on temperature was measured via photon correlation spectroscopy (N5 Submicron microparticle Size Analyzer, Beckman Coulter) at increasing temperature from 25°C to 45°C in MilliQ water (pH 5.5). The concentration of particles was determined by weighing the lyophilized particles.

### *T. cruzi* antigens and antibodies

In order to evaluate the ability of particles functionalized with different dyes to capture and concentrate *T. cruzi* antigens, we used: trypomastigote lysate antigen (TLA), a recombinant H49 antigen (kindly provided by Dr. Jose Franco da Silveira, Universidade Federal de São Paulo - São Paulo, Brazil), recombinant 1F8 *T. cruzi* antigen (Genway Biotech Inc, CA-USA), and trypomastigote excretory-secretory antigen (TESA). The TESA was harvested from cell cultures of *T. cruzi* Y strain in LLC-MK2 cells, as previously described [32]. For urine antigen detection we used a commercially available, monoclonal antibody against lipophosphoglycan (LPG) of *Trypanosoma cruzi* CL Brener strain (Cedarlane Laboratories, NC-USA), this antibody recognizes bands of 42 kDa and 82 kDa in TESA of *T. cruzi* Y strain.

### Pre analytical handling of urine samples and urinalysis

Urine samples of infants were collected after ingestion of milk. Sterile urine collection bags were maintained in position with adhesive until 5–10 ml of urine was collected or for a maximum of 1 hour. If this volume was not achieved within 1 hour, a new sterile bag was used. After collection samples were immediately centrifuged at 3000 relative centrifugal force (rcf) for 10 min and the supernatant was stored in liquid nitrogen or −70°C until use. The supernatant was adjusted to pH 5–6 with 1M HCl.

### Concentration of *T. cruzi* antigens in urine

Urine samples (5 mL) were incubated with 7.2 mg/ml of particles for 30 min at room temperature under rotation. After incubation, the samples were centrifuged at 16 000 rcf, 20 min at 25°C. Then, the particles were washed three times with MilliQ water by centrifugation at 16 000 rcf, 20 min, 25°C. Elution of the antigen from the particles was done using 300 µl of elution buffer (70% acetonitrile and 10% ammoniun hydroxide) for 10 min at room temperature, following by 5 minutes of sonication. The elution step was repeated twice and the eluates were pooled together. The eluates were dried under nitrogen flow (Microvap 118, Organomation Associates, Inc, MA-USA) at 40°C. The dried eluates were suspended in 40 µl of resuspending buffer {2500 µg trehalose (Fluka Chemicals, MO-USA) and 10 µl of 1% (v/v) red food dye (McCormick, MD-USA) in MilliQ water}; obtaining a concentration factor of 125 folds based on volume ratios (5000 µl/40 µl). For the Western Blot analysis 20 µl of resuspended antigens were analyzed. In each experiment we included a negative control (normal urine sample) and a positive control (5 ml of normal urine sample containing 1 ng of TESA antigen). The Chunap was carried out by a laboratory biologist who was also blinded to the Chagas status of the patient.

### Protection of antigens from degradation

In order to evaluate the ability of poly(NIPAm) particles to protect *T. cruzi* antigens from enzymatic degradation, H49 and 1F8 *T. cruzi* antigens (1 µg) were incubated with 3 ng of trypsin (Promega, WI, USA) at 37°C for one hour in 50 mM Tris-HCl pH 7.2, in the presence or absence of particles. After the incubation, antigens were eluted as described above and analyzed with SDS-PAGE analysis.

### Gel electrophoresis

SDS-PAGE analysis was performed using 4–20% Tris Glycine polyacrylamide gel (Invitrogen Corporation, CA-USA) using a Novex X-Cell IITM Mini-Cell (Invitrogen Corporation, CA-USA), at 200 V for 50 minutes. Visualization of bands was performed by silver staining or by Western blot.

### Detection of *T. cruzi* antigens by western blot

Resuspended antigens (20 µl) were mixed with 4 µl of sample buffer (50 mM TrisHCl pH 6.8 and treated with 2% SDS, 144 mM 2-mercaptoethanol, 10% glycerol and 0.01% bromophenol blue) and heated to 100°C for 7 min. The antigens were separated on 4–20% Tris Glycine polyacrylamide gels and then were transferred to polyvinylidene difluoride membranes (PVDF) (Millipore, MA-USA). The membranes were blocked with casein-based buffer: PBS supplemented with 0.1% Tween 20 (PBST) and 0.2% I-Block (Applied Biosciences, CA-USA) for one hour. The membranes were incubated overnight with mouse monoclonal antibody anti-LPG diluted 1/250 in the casein-based buffer. After six washing steps with PBST, membranes were incubated with peroxidase conjugated goat anti-mouse IgG and IgM (Invitrogen Corporation, CA-USA) diluted 1/5 000 in casein-based buffer for 60 minutes. Visualization of antigenic bands was done using an enhanced chemiluminescence system (Supersignal West Dura, Thermo Fisher Scientific, MA-USA). In each PVDF membrane we included 5 ml of normal urine sample containing 1 ng of TESA antigen and concentrated with of poly(NIPAm/TB) particles. For the Western blot each patient was run twice. The presence of any of the five diagnostic bands (22 kDa, 42 kDa, 58 kDa, 75 kDa and 82 kDa) was considered as a positive result for the Chunap. The criteria for defining a positive band depended on the judgment of a trained analyst.

### DNA extraction and real time PCR

Real time PCR was performed to evaluate levels of parasitemia in 500 µl of cord blood at birth or 200 µl of blood obtained at 1, 6 and 9 months-old. DNA extraction and quantitative real time PCR (qPCR) were performed based on published methods [33], [34] with the modifications detailed in a previous publication [7]. The primer set Cruzi 1 (5′-ASTCG-G-C-T-G-A-T-C-G-T-T-T-T-CGA-3′) and Cruzi 2 (5′-AAT-T-C-C-T-C-C-A-A-G-C-A-G-C-G-G-ATA-3′) was used to amplify a 166-base pair DNA fragment. The probe Cruzi 3 (5′-CAC-A-C-A-C-T-G-G-A-C-A-C-CAA-3′) was labeled with 5′FAM (6-carboxyfluorescein) and 3′MGB (minor groove binder).

### Statistical analysis

STATA 10.0 software was used to calculate the sensitivity and specificity of each diagnostic test with 95% confidence interval.

## Results

### Selection of optimal protein affinity bait for concentration of *T. cruzi* antigens

**Figure 1:**
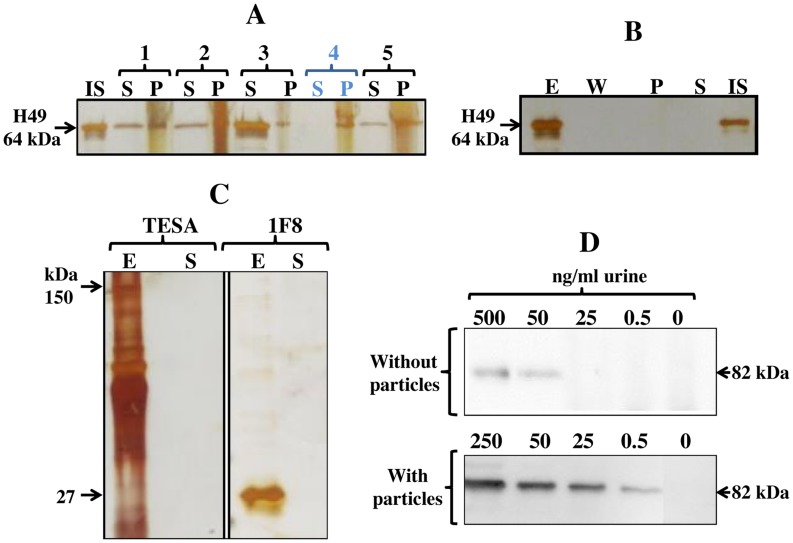
Sequestration and concentration of *T. cruzi* antigens by bait functionalized poly(NIPAm) particles. Silver stain analysis: A. H49 antigen (50 ng) was spiked in 250 µl of normal urine samples, and incubated with different NIPAm particles for 15 min at room temperature. Particles were separated by centrifugation, and a SDS-PAGE analysis was performed of the particles (containing bound proteins) and the supernatant (20 µl, containing unbound proteins). NIPAm based particles functionalized with different affinity baits successfully captured and concentrated *T. cruzi* antigens (H49 recombinant protein). Poly(NIPAm/trypan blue) (TB) particles completely captured H49 antigen and deplete the supernatant. IS: Initial Solution. S: Supernatant (unbound proteins). P: Particles (containing bound proteins). B. 250 µl of H49 antigen (20 ng) was incubated with poly (NIPAm/trypan blue) particles. After incubation and centrifugation the supernatant (S, unbound proteins) was saved and particles were washed with 250 µl of miliQ water. After centrifugation, the wash solution (W) was saved and the elution of antigens from particles was performed using acetonitrile-based elution buffer (E). A complete elution of H49 antigen from poly(NIPAm/TB) particles was obtained, and *T. cruzi* antigens were not lost during the washing step. E: Elute, W: washing solution (20 µl), P: Particle content after elution indicating not presence of H49 antigen, S: supernatant (20 µl, unbound proteins), IS: Initial Solution (20 µl, corresponds to 1.6 ng). C. Poly(NIPAm/TB) particles completely capture different types of *T. cruzi* antigens. TESA: Trypomastigote excretory-secretory antigen and 1F8: recombinant antigen 1F8. E: Elute from particles. S: Supernatant (unbound proteins). Western Blot analysis using a mouse monoclonal antibody to LPG of *T. cruzi*: D. Concentration of trypomastigote lysate antigen (TLA) in urine using poly(NIPAm/TB) particles. Poly(NIPAm/TB) particles capture and concentrate TLA antigen in the presence of excess competing proteins in urine. The limit of detection of TLA antigen by Western Blot substantially improves when urine samples were treated with particles, from 50 ng/ml without particle treatment to 0.5 ng/ml with particle treatment.

Poly (N-isopropyl acrylamide) (NIPAm) particles functionalized with all molecular baits tested in this study (acid blue 22, acid black 48, bismarck brown Y, pararosaniline base, trypan blue and remazol brilliant blue R) captured H49 and TLA antigens to some degree; but poly(NIPAm) particles functionalized with trypan blue [poly(NIPAm/TB)] particles were the most effective because they completely sequestered the target protein from the solution (Figure 1A). In order to further characterize the yield of poly(NIPAm/TB) particles pre-processing step, we demonstrated by SDS PAGE analysis that H49 antigen was not lost during the washing step and was eluted from the particles with a yield higher than 95% (Figure 1B) (similar results were obtained for TLA). In order to investigate whether poly(NIPAm/TB) particles have high affinity also for other *T. cruzi* antigens, particles were incubated with TESA and recombinant 1F8 antigen. SDS PAGE analysis demonstrated that poly(NIPAm/TB) particles completely sequestered all the *T. cruzi* antigens tested (Figure 1C).

Our Chagas urine nanoparticle test (Chunap) uses poly(NIPAm/TB) particles for concentration of *T. cruzi* antigens in urine. A detection limit (DL) of 0.5 ng/ml was achieved with an initial urine volume of 5 milliliters after concentration of antigens by poly (NIPAm/TB) particles. The DL of western blot without particle concentration preprocessing step was 50 ng/ml, yielding a concentration factor of 100 fold (Figure 1D).

### Poly(NIPAm/trypan blue) particles characterization

Hydrogel particles hydrodynamic dimensions typically exhibit a temperature responsive behavior. The diameter of the poly (NIPAm/TB) particles decreased with increasing temperature, as expected (from 780 nm at 20°C to 320 nm at 45°C). The diameter of particles at 25°C and pH 4.5 was 758.6 nm±15.03.

### Protection from degradation of *T. cruzi* antigens by poly (NIPAm)/trypan blue particles

**Figure 2:**
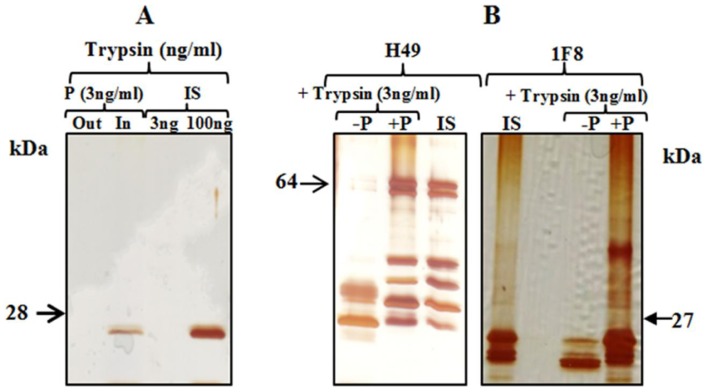
Protection from degradation of recombinant H49 and 1F8 *T. cruzi* antigens by poly(NIPAm/Trypan Blue) particles. A. Poly(NIPAm/trypan blue) particles capture and concentrate trypsin in spiked normal urine samples. P: Particles (trypsin: 3 ng/ml); Out: Outside the particles (unbound proteins); In: Inside the particles (bound proteins)); IS: Initial Solution. B. H49 and 1F8 *T. cruzi* antigens spiked in normal urine samples are protected from trypsin digestion by poly(NIPAm/trypan blue) particles. −P: *T. cruzi* antigen and trypsin without particles. + P: *T. cruzi* antigen and trypsin with particles. IS: Initial Solution of *T. cruzi* antigen without trypsin.

Bait functionalized capturing particles protect captured analytes from enzymatic degradation even if the degradative enzyme is small enough to penetrate inside the particles [24]. In this study, trypsin was captured and concentrated by poly(NIPAm/TB) particles (Figure 2A). Even if trypsin was fully captured by poly (NIPAm/TB) particles, H49 and 1F8 *T. cruzi* antigens were completely protected from enzymatic digestion in the presence of poly(NIPAm)/TB particles (Figure 2B). As a positive control, complete degradation of H49 and 1F8 *T. cruzi* antigens was observed in presence of trypsin at 37°C after 1 hour (Figure 2B).

### Detection of *T. cruzi* antigens in urine of infants

**Figure 3:**
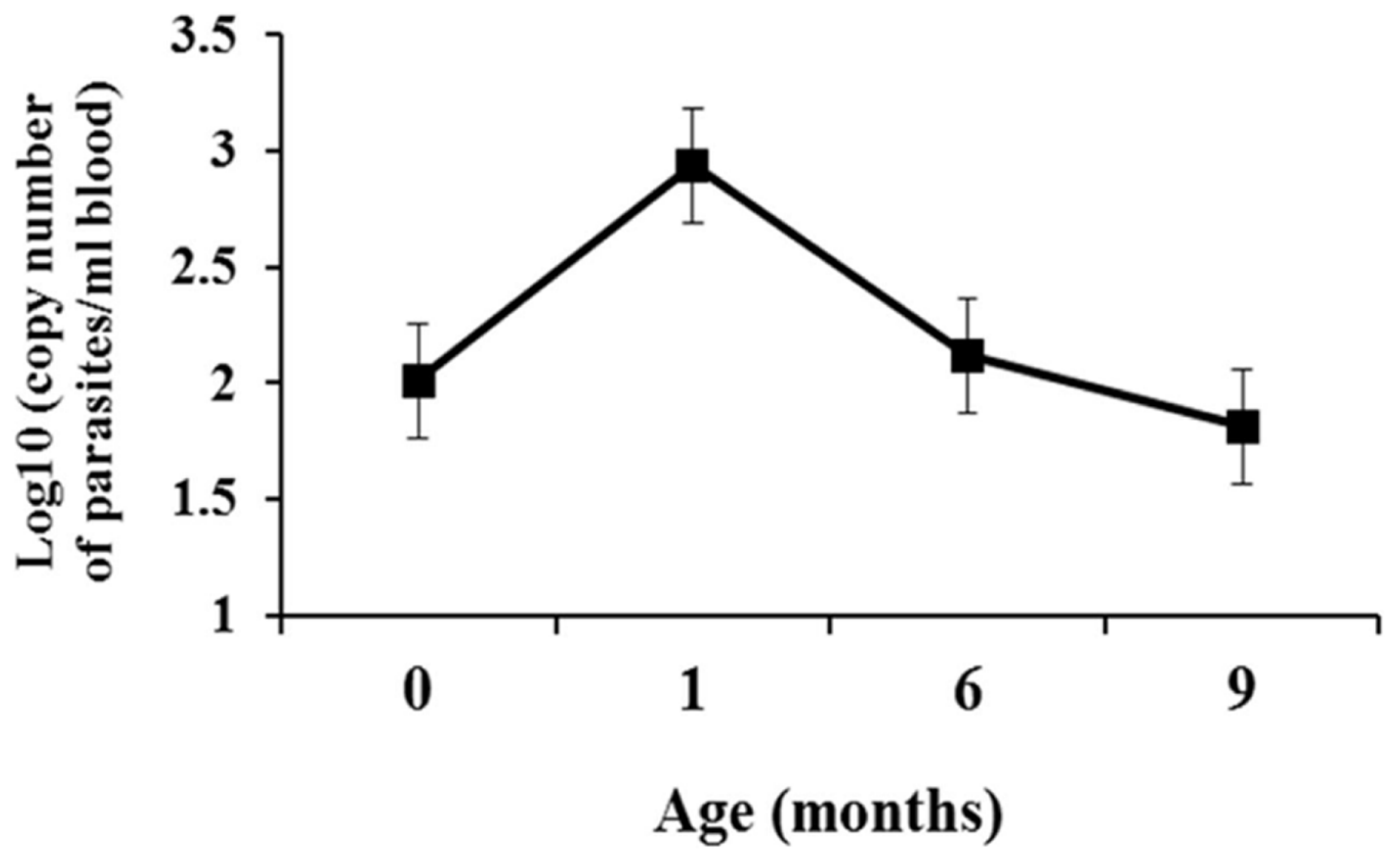
Levels of parasitemia in infants congenitally infected with *T. cruzi*. PCR targeting *T. cruzi* kinetoplast DPT was done from blood from umbilical cord at birth or blood samples. Number of individuals per group of age: 0 months: n = 9; 1 month: n = 10; 6 months: n = 6; 9 months: n = 4.

The table 1 shows the results of diagnostic testing for each infected infant. Combining the results from the birth and 1-month specimens, the cumulative sensitivity of micromethod and PCR was 34.8% (8/23) and 91.3% (21/23), respectively. Bands of 22 kDa, 42 kDa, 58 kDa, 75 kDa and 82 kDa were detected in urine samples of infected babies (Figure S2). The presence of any of these five bands was considered as a positive result for the Chunap. Chunap showed 91.3% sensitivity (95% CI: 71.92%–98.68%) in a single specimen at one month of age, comparable to the 88.2% sensitivity of PCR at this time point (95% CI: 63.5%–98.2%) (Table 1). Parasitemia levels determined by qPCR peaked at one month of age with subsequent decrease over time (Figure 3). Chunap specificity was 96.5% (95% CI: 90.1% to 99.2%, 71 negative results/74 specimens from uninfected babies in the endemic site, 12 negative results/12 specimens from babies in the non-endemic site).

**Table 1:** Diagnostic test results in infants with congenital *T. cruzi* infection.

	Micromethod		PCR		Serology	Chunap	
Infant code	Birth	1 month	Birth	1 month	6 to 12 months	Chunap 1 month	Chunap Bands
1	NEG	NEG	**POS**	**POS**	**POS**	**POS**	22 kDa, 75 kDa
2	NEG	NEG	NEG	**POS**	**POS**	**POS**	22 kDa, 75 kDa
3	NEG	**POS**	**POS**	**POS**	PT	**POS**	22 kDa
4	NEG	NEG	NEG	NEG	**POS**	**POS**	22 kDa, 58 kDa
5	NEG	NEG	**POS**	**POS**	**POS**	**POS**	82 kDa
6	NEG	NEG	NEG	**POS**	**POS**	**POS**	42 kDa
7	**POS**	PT	**POS**	**POS**	PT	**POS**	42 kDa
8	**POS**	PT	**POS**	PT	PT	**POS**	42 kDa
9	**POS**	PT	NEG	**POS**	PT	**POS**	22 kDa, 58 kDa
10	NEG	**POS**	NEG	**POS**	PT	**POS**	22 kDa, 42 kDa, 58 kDa
11	NEG	NEG	**POS**	**POS**	**POS**	**POS**	22 kDa, 58 kDa
12	NEG	**POS**	NEG	**POS**	PT	**POS**	22 kDa, 58 kDa
13	**POS**	PT	**POS**	PT	PT	**POS**	82 kDa
14	NEG	NEG	**POS**	**POS**	**POS**	**POS**	82 kDa
15	NEG	NEG	NEG	**POS**	**POS**	**POS**	82 kDa
16	NEG	NEG	**POS**	no data	**POS**	**POS**	82 kDa
17	NEG	NEG	**POS**	**POS**	**POS**	**POS**	22 kDa, 58 kDa
18	NEG	**POS**	**POS**	no data	PT	**POS**	22 kDa, 58 kDa, 75 kDa, 82 kDa
19	NEG	NEG	**POS**	no data	**POS**	**POS**	22 kDa, 42 kDa, 58 kDa, 82 kDa
20	NEG	NEG	no data	NEG	**POS**	**POS**	22 kDa, 42 kDa, 58 kDa, 82 kDa
21	NEG	NEG	NEG	**POS**	**POS**	**POS**	22 kDa, 58 kDa, 75 kDa, 82 kDa
22	NEG	NEG	NEG	**POS** 1	**POS**	NEG	
23	NEG	NEG	**POS**	no data	**POS**	NEG	
**Total**	**4/23**	**4/19**	**13/22**	**15/17**	**15/15**	**21/23**	
**Sensitivity (%)**	**17**	**21**	**59**	**88**	**100**	**91**	
**(95% CI)**	(5.1–38.8)	(6.2–45.6)	(36.4–79.3)	(63.5–98.2)	(78.0–100.0)	(71.9–98.7)	

The results of each test are reported only if the samples were obtained before the treatment was initiated.

1 Specimen taken at 3 months.

95% CI: 95% Confidential Interval.

PT: Post-treatment.

POS: Positive. NEG: Negative.

## Discussion

In this study, we demonstrate for the first time that our Chagas urine nanoparticle assay (Chunap) detects congenital Chagas disease in a single urine specimen at one month of life with more than 90% sensitivity and more than 95% specificity. The study also shows that poly(NIPAm) particles coupled with trypan blue dye efficiently capture and concentrate *T. cruzi* antigens in urine, and under experimental conditions these particles protect *T. cruzi* antigens in urine from enzymatic degradation. Evaluation at one month of age provides high sensitivity because this time point is characterized by the highest levels of parasitemia and therefore also excretion of high levels of antigen. Nanotechnology-based tests can be adapted to point-of-care and cost-effective detection of microbial agents, as shown for other infectious diseases [35].The non-invasive nature of the test will also greatly enhance parental acceptability.

We evaluated the performance of poly(NIPAm) particles coupled with five different high affinity dyes. Optimal results were obtained with poly (NIPAm) particles with trypan blue (TB), which achieved a 100-fold increase in antigen concentration in urine. Our data demonstrate that these nanoparticles can capture and concentrate *T. cruzi* analytes of different chemical structures, including proteins (H49 and 1F8), glycoproteins (TESA) and lipophosphoglycan. The broad range of antigens captured provides an advantage over other methodologies that target a specific chemical structure [20], enabling the use of these nanoparticles as a single pre-processing step for sensitive multiplex analysis of several urinary analytes simultaneously.

Bait-loaded hydrogel nanoparticles also preserve captured proteins from enzymatic degradation, even when the proteolytic enzyme (e.g. trypsin, as in this study) is small enough to penetrate inside the particles. We hypothesize that the mechanism of protection stems from the immobilization of trypsin by the nano-porous particle, which prevents the enzyme from binding substrate proteins. Another possibility is the steric hindrance associated with trapping of the substrate by the affinity-bait groups in the particle, which may prevent enzymes from productively binding target proteins [24]. Antigens in urine can be potentially degraded in the urinary track or bladder; however, studies indicate that this degradation is minimal [36]. Degradation of antigens after urine collection is enhanced due to bacterial contamination, so that the advantage of hydrogel particles is their protective effect immediately after urine collection. Further studies using urine samples collected at different times (first morning urine vs random spot urine collection) may help evaluate the extent of degradation in the urinary tract or bladder. However, random spot urine collection makes the test simpler [37].

Two previous studies have utilized urine antigen detection to diagnose congenital Chagas, reporting sensitivities of 80%–100% [20], [21]. However, these studies had small sample sizes (n = 10 and 14) and all but two of the congenital cases had parasitemia detected by microscopy. By contrast, nearly two-thirds of our infected infants were missed by micromethod, implying lower levels of parasitemia.

In this study, PCR showed good sensitivity early in life (91.3% when results from birth and 1-month specimens were combined), as previously reported by other studies [7], [9]. Two infants had positive results by Chunap but negative results by PCR; the large volume concentrated by the hydrogel nanoparticles may enable detection in some cases when antigen loads in the urine are low. Similar to qPCR, antigenuria also has the advantage of permitting early treatment which is associated with higher cure rates and fewer side effects compared to treatment later in life. Early diagnosis also translates to a much lower rate of missed infections compared to an algorithm requiring 6–12 months of follow-up [7], [15].

To our knowledge, this study is the first to successfully and consistently detect *T. cruzi* LPG in urine of Chagas infected infants and to use this analyte for diagnostic purposes. Similarly, LPG of *Leishmania* has been detected in urine of patients [38]. LPG has been shown to play a key role in host-cell recognition/invasion and in parasite survival. LPG is highly expressed in *T. cruzi* with a cellular copy number of 4×l0^5^ molecules of LPG glycoconjugates/cell making it a good candidate for diagnosis [39] and a monoclonal antibody is commercially available. The monoclonal antibody that we used was directed against the LPG of *T. cruzi* CL Brener strain, corresponding to the hybrid genotype VI [40]. This antibody also recognizes LPG fractions (band of 82 kDa) in TLA and TESA of *T. cruzi* Y strain (genotype II) and of a genotype I strain isolated from a patient from Bolivia (Figure S3), suggesting that this antibody can identify LPG preparations of most strains of *T. cruzi*. However, further studies must be performed in order to determine the ability of this antibody to recognize the LPG of other genotypes.

With appropriate adaptation to a field-friendly format, Chunap has the potential to enable early point-of-care diagnosis of congenital Chagas disease in peripheral health facilities. Further steps will be necessary to apply this nanotechnology in developing countries. We are currently optimizing a novel separation method based on magnetic labeling of capturing nanoparticles to enable particle separation from urine without the need for a high speed centrifuge. Finally, although used in this study for Chagas disease, this method could be adapted for detection of other parasitic infections in urine and other body fluids.

## Supporting Information

Checklist S1 **“Use of a Novel Chagas Urine Nanoparticle Test (Chunap) for Diagnosis of Congenital Chagas Disease.”**
(DOC)

Figure S1 **Flow diagram of the study.** “n” represents the number of individuals in each group.
(TIF)

Figure S2 **Detection of** ***T. cruzi*** **antigens in nanoparticles-concentrated urine samples of infants.** Bands of 22 kDa, 42 kDa, 58 kDa, 75 kDa and 82 kDa were detected by Western Blot using a mouse monoclonal antibody against lipophosphoglycan of *T. cruzi*. Lanes 1–21: Patient codes of infants with congenital *T. cruzi* infection (See Table 1 for more details). Ns: Infants without congenital *T. cruzi* infection.
(TIF)

Figure S3 **Detection of lipophosphoglycan in trypomastigote excretory-secretory antigen (TESA) of** ***T. cruzi*** **Bolivia and Y strains.** A. Two bands of 42 kDa and 82 kDa were detected by Western Blot using a monoclonal antibody to lipophosphoglycan of *T. cruzi* CL Brener strain (genotype VI). 1. *T. cruzi* Bolivia strain (genotype I). 2. *T. cruzi* Y strain (genotype II). B. Periodic acid–Schiff stain demonstrating the polysaccharide content of the 82 kDa band of *T. cruzi* Bolivia strain (lane 1) and Y strain (lane 2).
(TIF)

## References

[1] Rassi A Jr, Rassi A, Marin-Neto JA (2010) Chagas disease. Lancet 375 9723: 1388–402. 20399979 10.1016/S0140-6736(10)60061-X

[2] Organización Panamericana de la Salud (2006) Estimación cuantitativa de la enfermedad de Chagas en las Américas. Montevideo, Uruguay: Organización Panamericana de la Salud.

[3] World Health Assembly (2010) Chagas disease: control and elimination. In: Sixty-third World Health Assembly Resolutions, Geneva. Resolutions and decisions, annexes (WHA63/2010/REC/1), resolution WHA63.20:39–42.

[4] Voelker R (2012) Congenital Chagas disease reported in United States. JAMA 308 5: 443. 22851094 10.1001/jama.2012.9468

[5] Schenone H, Gaggero M, Sapunar J, Contreras MC, Rojas A, et al (2001) Congenital Chagas disease of second generation in Santiago, Chile. Report of two cases. Rev Inst Med Trop Sao Paulo 43 4: 231–2. 11558005 10.1590/s0036-46652001000400011

[6] Blanco SB, Segura EL, Cura EN, Chuit R, Tulián L, et al (2000) Congenital transmission of *Trypanosoma cruzi*: an operational outline for detecting and treating infected infants in north-western Argentina. Trop Med Int Health 5 4: 293–301. 10810029 10.1046/j.1365-3156.2000.00548.x

[7] Bern C, Verastegui M, Gilman RH, Lafuente C, Galdos-Cardenas G, et al (2009) Congenital *Trypanosoma cruzi* transmission in Santa Cruz, Bolivia. Clin Infect Dis 49 11: 1667–74. 19877966 10.1086/648070 PMC5454522

[8] Mora MC, Sanchez Negrette O, Marco D, Barrio A, Ciaccio M, et al (2005) Early diagnosis of congenital *Trypanosoma cruzi* infection using PCR, hemoculture, and capillary concentration, as compared with delayed serology. J Parasitol 91 6: 1468–73. 16539033 10.1645/GE-549R.1

[9] Bern C, Martin D, Gilman RH (2011) Acute and congenital Chagas disease. Advances in Parasitology 75: 19–47. 21820550 10.1016/B978-0-12-385863-4.00002-2

[10] Segura EL, Esquivel ML, Salomón O, Gómez AO, Sosa Estani S, et al (1994) Community participation in the National Program for Transmission Control of Chagas Disease. Medicina (B Aires) 54 5 Pt 2: 610–1. 7658995

[11] Blanco SB, Segura EL, Gürtler RE (1999) Control of congenital transmission of *Trypanosoma cruzi* in Argentina. Medicina (B Aires) 59 Suppl 2: 138–42. 10668256

[12] Oliveira I, Torrico F, Muñoz J, Gascon J (2010) Congenital transmission of Chagas disease: a clinical approach. Expert Rev Anti Infect Ther 8 8: 945–56. 20695749 10.1586/eri.10.74

[13] Sánchez Negrette O, Mora MC, Basombrío MA (2005) High prevalence of congenital *Trypanosoma cruzi* infection and family clustering in Salta, Argentina. Pediatrics 115 6: e668–72. 15930194 10.1542/peds.2004-1732

[14] Romero M, Postigo J, Schneider D, Chippaux JP, Santalla JA, et al (2011) Door-to-door screening as a strategy for the detection of congenital Chagas disease in rural Bolivia. Trop Med Int Health 16 5: 562–9. 21342373 10.1111/j.1365-3156.2011.02746.x

[15] Cardoso EJ, Valdéz GC, Campos AC, de la Luz Sanchez R, Mendoza CR, et al (2012) Maternal fetal transmission of *Trypanosoma cruzi*: a problem of public health little studied in Mexico. Exp Parasitol 131 4: 425–32. 22683499 10.1016/j.exppara.2012.05.013

[16] Carlier Y, Torrico F, Sosa-Estani S, Russomando G, Luquetti A, et al (2011) Congenital Chagas disease: recommendations for diagnosis, treatment and control of newborns, siblings and pregnant women. PLoS Negl Trop Dis 5 10: e1250. 22039554 10.1371/journal.pntd.0001250 PMC3201907

[17] Howard EJ1, Xiong X, Carlier Y, Sosa-Estani S, Buekens P (2013) Frequency of the congenital transmission of *Trypanosoma cruzi*: a systematic review and meta-analysis. BJOG 121 1: 22–33. 23924273 10.1111/1471-0528.12396 PMC3914719

[18] Umezawa ES, Shikanaiyasuda MA, Dasilveira JF, Cotrim PC, Paranhos G, et al (1993) *Trypanosoma cruzi*: Detection of a Circulating Antigen in Urine of Chagasic Patients Sharing Common Epitopes with an Immunodominant Repetitive Antigen. Experimental Parasitology 76: 352–357. 7685708 10.1006/expr.1993.1043

[19] Castro-Sesquen YE, Gilman RH, Yauri V, Cok J, Angulo N, et al (2013) Detection of soluble antigen and DNA of *Trypanosoma cruzi* in urine is independent of renal injury in the guinea pig model. PLoS One 8 3: e58480. 23520515 10.1371/journal.pone.0058480 PMC3592799

[20] Corral RS, Altcheh J, Alexandre SR, Grinstein S, Freilij H, et al (1987) Detection and characterization of antigens in urine of patients with acute, congenital, and chronic Chagas' disease. J Clin Microbiol 34 8: 1957–62. 10.1128/jcm.34.8.1957-1962.1996 PMC229162 8818890

[21] Freilij HL, Corral RS, Katzin AM, Grinstein S (1987) Antigenuria in infants with acute and congenital Chagas' disease. J Clin Microbiol 25 1: 133–7. 3098778 10.1128/jcm.25.1.133-137.1987 PMC265840

[22] Katzin A, Manso M, Abuin' G, CoIli W (1989) Antigenuria in chronic chagasic patients detected by a monoclonal antibody raised against *Trypanosoma cruzi* . Trans R Soc Trop Med Hyg 83: 341–343. 2482556 10.1016/0035-9203(89)90497-5

[23] Luchini A, Longo C, Espina V, Petricoin EF 3rd, Liotta LA (2010) Nanoparticle technology: addressing the fundamental roadblocks to protein biomarker discovery. Curr Mol Med 10 2: 133–41. 20196732 10.2174/156652410790963268 PMC2873152

[24] Luchini A, Geho DH, Bishop B, Tran D, Xia C, et al (2008) Smart hydrogel particles: biomarker harvesting: one-step affinity purification, size exclusion, and protection against degradation. Nano letters 8 1: 350–61. 18076201 10.1021/nl072174l PMC2877922

[25] Douglas TA, Tamburro D, Fredolini C, Espina BH, Lepene BS, et al (2010) The use of hydrogel microparticles to sequester and concentrate bacterial antigens in a urine test for Lyme disease. Biomaterials 32 4: 1157–1166. 21035184 10.1016/j.biomaterials.2010.10.004 PMC3019571

[26] Fredolini C, Meani F, Reeder KA, Rucker S, Patanarut A, et al (2008) Concentration and Preservation of Very Low Abundance Biomarkers in Urine, such as Human Growth Hormone (hGH), by Cibacron Blue F3G-A Loaded Hydrogel Particles. Nano Res 1 6: 502–518. 20467576 10.1007/s12274-008-8054-z PMC2868260

[27] Longo C, Patanarut A, George T, Bishop B, Zhou W, et al (2009) Core-shell hydrogel particles harvest, concentrate and preserve labile low abundance biomarkers. PLoS One 4 3: e4763. 19274087 10.1371/journal.pone.0004763 PMC2651577

[28] Tamburro D, Fredolini C, Espina V, Douglas TA, Ranganathan A, et al (2011) Multifunctional core-shell nanoparticles: discovery of previously invisible biomarkers. J Am Chem Soc 133 47: 19178–88. 21999289 10.1021/ja207515j PMC3223427

[29] Verani JR, Seitz A, Gilman RH, LaFuente C, Galdos-Cardenas G, et al (2009) Geographic variation in the sensitivity of recombinant antigen-based rapid tests for chronic *Trypanosoma cruzi* infection. Am J Trop Med Hyg 80 3: 410–5. 19270291

[30] Freilij H, Muller L, Gonzalez Cappa SM (1983) Direct micromethod for diagnosis of acute and congenital Chagas' disease. J Clin Micro 18: 327–330. 10.1128/jcm.18.2.327-330.1983 PMC270800 6413530

[31] Programa Nacional de Control de Chagas (2007) Chagas Congénito: Estrategias de Diagnóstico y Control. In. 2nd ed: Digital Dreams, Cochabamba, Bolivia: 1–89.

[32] Umezawa ES, Nascimento MS, Kesper N Jr, Coura JR, Borges-Pereira J, et al (1996) Immunoblot assay using excreted-secreted antigens of *Trypanosoma cruzi* in serodiagnosis of congenital, acute, and chronic Chagas' disease. J Clin Microbiol 34 9: 2143–7. 8862574 10.1128/jcm.34.9.2143-2147.1996 PMC229206

[33] Fitzwater S, Calderon M, Lafuente C, Galdos-Cardenas G, Ferrufino L, et al (2008) Polymerase chain reaction for chronic *Trypanosoma cruzi* infection yields higher sensitivity in blood clot than buffy coat or whole blood specimens. Am J Trop Med Hyg 79 5: 768–70. 18981520

[34] Piron M, Fisa R, Casamitjana N, López-Chejade P, Puig L, et al (2007) Development of a real-time PCR assay for *Trypanosoma cruzi* detection in blood samples. Acta Trop 103 3: 195–200. 17662227 10.1016/j.actatropica.2007.05.019

[35] Syed MA (2014) Advances in nanodiagnostic techniques for microbial agents. Biosens Bioelectron 51: 391–400. 24012709 10.1016/j.bios.2013.08.010

[36] Zhou H, Yuen PS, Pisitkun T, Gonzales PA, Yasuda H, et al (2006) Collection, storage, preservation, and normalization of human urinary exosomes for biomarker discovery. Kidney Int 69 8: 1471–6. 16501490 10.1038/sj.ki.5000273 PMC2276656

[37] Thomas CE, Sexton W, Benson K, Sutphen R, Koomen J (2010) Urine collection and processing for protein biomarker discovery and quantification. Cancer Epidemiol Biomarkers Prev 19 4: 953–9. 20332277 10.1158/1055-9965.EPI-10-0069 PMC2852495

[38] Sarkari B, Chance M, Hommel M (2002) Antigenuria in visceral leishmaniasis: detection and partial characterisation of a carbohydrate antigen. Acta Trop 82 3: 339–48. 12039673 10.1016/s0001-706x(02)00043-8

[39] Singh BN, Lucas JJ, Beach DH, Costello CE (1994) Expression of a novel cell surface lipophosphoglycan-like glycoconjugate in *Trypanosoma cruzi* epimastigotes. J Biol Chem 269 35: 21972–82. 8071317

[40] Zingales B, Andrade SG, Briones MRS, Campbell DA, Chiari E, et al (2009) A new consensus for *Trypanosoma cruzi* intraspecific nomenclature: second revision meeting recommends TcI to TcVI. Mem Inst Oswaldo Cruz 104 7: 1051–1054. 20027478 10.1590/s0074-02762009000700021

