# Supplementary figures and images for: Use of a Novel Chagas Urine Nanoparticle Test (Chunap) for Diagnosis of Congenital Chagas Disease
Source: PLoS Negl Trop Dis. 2014 Oct 2;8(10):e3211. doi: 10.1371/journal.pntd.0003211 (PMC4183489; doi:10.1371/journal.pntd.0003211)

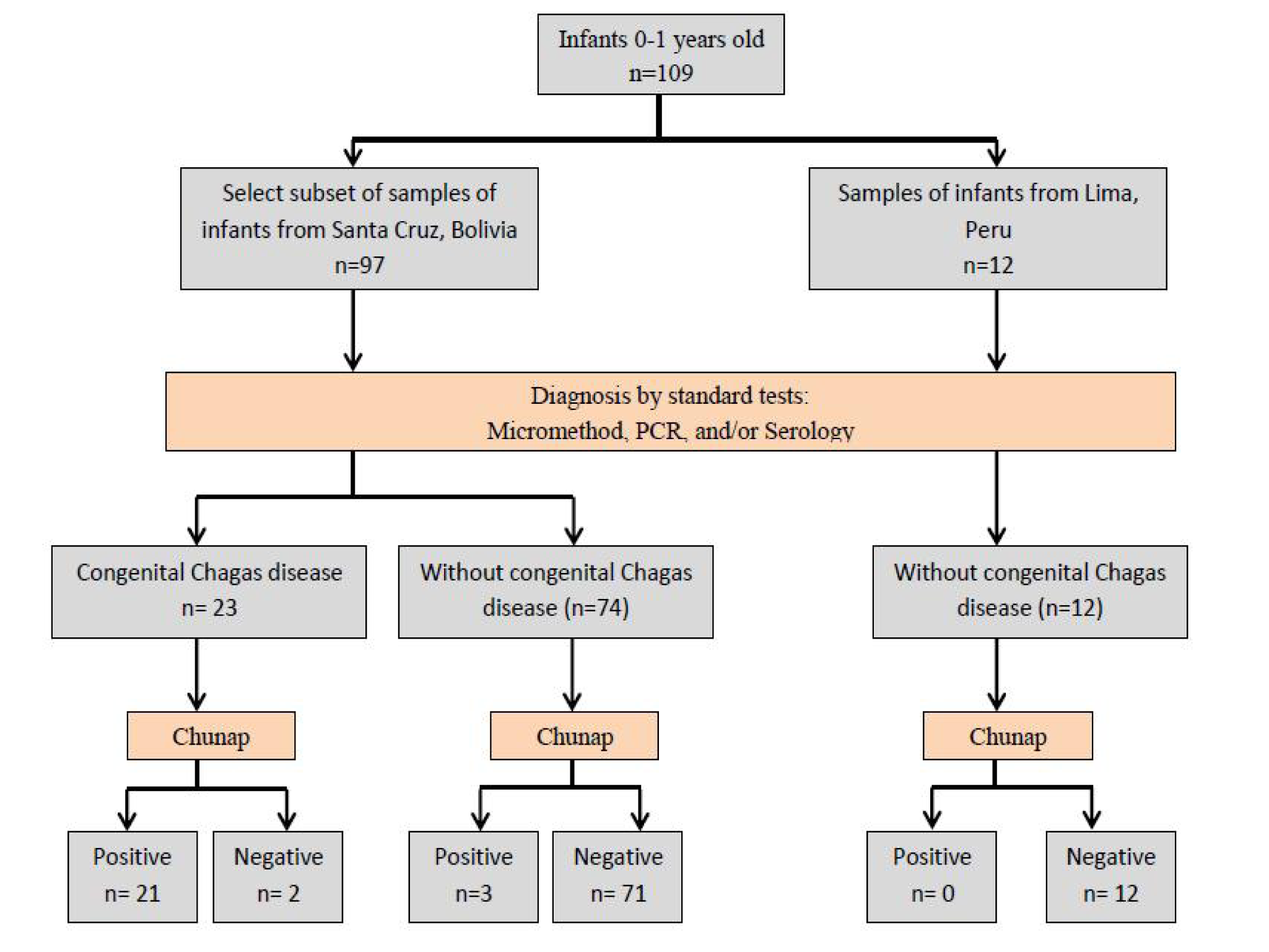

Supplement: Figure S1 — Flow diagram of the study. “n” represents the number of individuals in each group. (TIF) [file pntd.0003211.s002.tif]

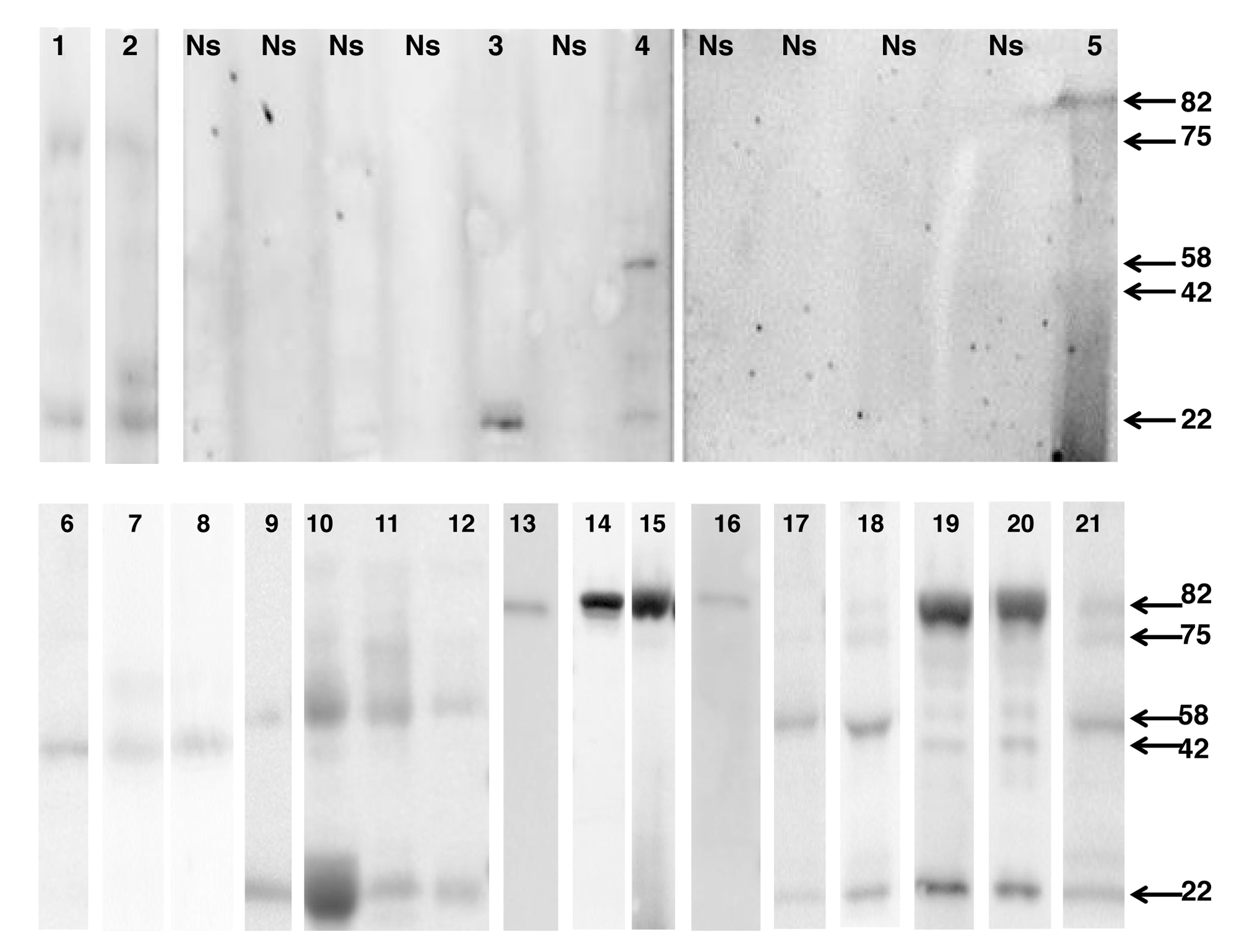

Supplement: Figure S2 — Detection of T. cruzi antigens in nanoparticles-concentrated urine samples of infants. Bands of 22 kDa, 42 kDa, 58 kDa, 75 kDa and 82 kDa were detected by Western Blot using a mouse monoclonal antibody against lipophosphoglycan of T. cruzi. Lanes 1–21: Patient codes of infants with congenital T. cruzi infection (See Table 1 for more details). Ns: Infants without congenital T. cruzi infection. (TIF) [file pntd.0003211.s003.tif]

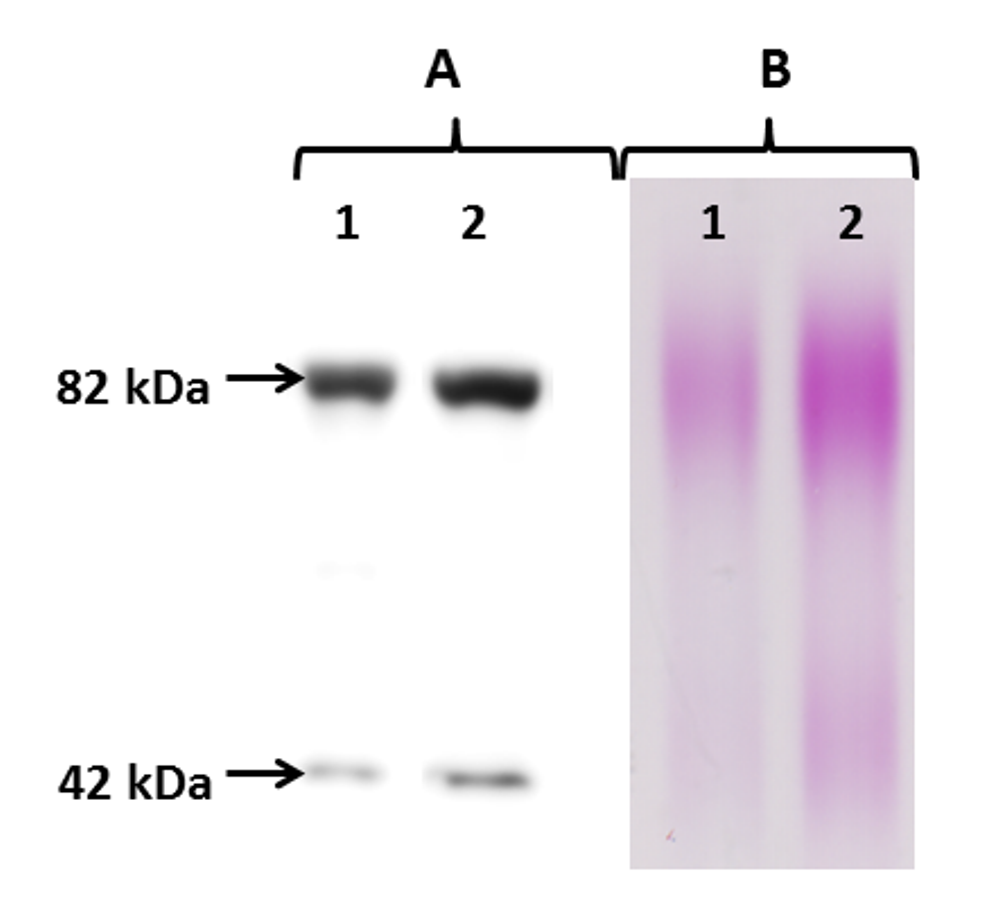

Supplement: Figure S3 — Detection of lipophosphoglycan in trypomastigote excretory-secretory antigen (TESA) of T. cruzi Bolivia and Y strains. A. Two bands of 42 kDa and 82 kDa were detected by Western Blot using a monoclonal antibody to lipophosphoglycan of T. cruzi CL Brener strain (genotype VI). 1. T. cruzi Bolivia strain (genotype I). 2. T. cruzi Y strain (genotype II). B. Periodic acid–Schiff stain demonstrating the polysaccharide content of the 82 kDa band of T. cruzi Bolivia strain (lane 1) and Y strain (lane 2). (TIF) [file pntd.0003211.s004.tif]
